# Testing Jump-Diffusion in Epileptic Brain Dynamics: Impact of Daily Rhythms

**DOI:** 10.3390/e23030309

**Published:** 2021-03-05

**Authors:** Jutta G. Kurth, Thorsten Rings, Klaus Lehnertz

**Affiliations:** 1 Department of Epileptology, University Hospital Bonn, Venusberg Campus 1, 53127 Bonn, Germany; Jutta.G.Kurth@web.de (J.G.K.); Thorsten.Rings@uni-bonn.de (T.R.); 2 Helmholtz Institute for Radiation and Nuclear Physics, University of Bonn, Nussallee 14-16, 53115 Bonn, Germany; 3 Interdisciplinary Center for Complex Systems, University of Bonn, Brühler Straße 7, 53175 Bonn, Germany

**Keywords:** diffusion process, jump-diffusion process, time series analysis, brain, epilepsy, biological rhythms

## Abstract

Stochastic approaches to complex dynamical systems have recently provided broader insights into spatial-temporal aspects of epileptic brain dynamics. Stochastic qualifiers based on higher-order Kramers-Moyal coefficients derived directly from time series data indicate improved differentiability between physiological and pathophysiological brain dynamics. It remains unclear, however, to what extent stochastic qualifiers of brain dynamics are affected by other endogenous and/or exogenous influencing factors. Addressing this issue, we investigate multi-day, multi-channel electroencephalographic recordings from a subject with epilepsy. We apply a recently proposed criterion to differentiate between Langevin-type and jump-diffusion processes and observe the type of process most qualified to describe brain dynamics to change with time. Stochastic qualifiers of brain dynamics are strongly affected by endogenous and exogenous rhythms acting on various time scales—ranging from hours to days. Such influences would need to be taken into account when constructing evolution equations for the epileptic brain or other complex dynamical systems subject to external forcings.

## 1. Introduction

The human brain’s structural and functional complexity [1,2,3] make it one of the most complicated and fascinating dynamical systems in nature. It is a complex network of interacting non-stationary subsystems, whose complicated spatial-temporal dynamics is still poorly understood. This holds true particularly for the case of brain pathologies with coexisting normal and abnormal functions and/or structures. A prominent example is epilepsy along with its cardinal symptom—epileptic seizures. Epilepsy is one of the most common neurological diseases globally, affecting an estimated 50 million people worldwide [4]. Seizures cannot sufficiently be controlled pharmacologically in approximately 30% of people with epilepsy [5], and the exact mechanism underlying the generation of seizures and related pathophysiological activities in humans are not fully understood.

Over the last decades nonlinear dynamics theory has contributed significantly to improve the understanding of epileptic brain dynamics [6,7,8]. Applying nonlinear time series analysis techniques [9] to electroencephalographic data (EEG) recorded in subjects with epilepsy provided strong evidence that the epileptic process appears as a nonlinear deterministic dynamics in an otherwise stochastic environment [10,11,12]. A more detailed characterization of spatial and temporal aspects of the epileptic process can be achieved with stochastic qualifiers [13,14,15,16] that are based on specific aspects of the (higher order) Kramers-Moyal (KM) coefficients, which can be derived from time series data [17,18]. Findings so far achieved with this approach indicate that crucial aspects of pathological brain dynamics must be regarded as a high-dimensional stochastic process in many cases. Moreover, they also indicate the high suitability of generalizing the Langevin-type modeling to a jump-diffusion modeling to further improve the characterization of pathological brain dynamics beyond a continuous process [16].

The results reported in Ref. [16], however, also reveal that a clear cut distinction between physiological and pathophysiological activities from the seizure-free interval along with their spatial extent appears to be impacted by other influencing factors not yet taken into account. To gain further insights into this issue, we estimate in a time-resolved manner KM coefficients up to order 6 from exemplary EEG time series that were recorded from a subject with epilepsy. We then make use of a recently proposed criterion that allows one to check whether for a given, even noisy time series the underlying process has a continuous (diffusion) trajectory or has jump discontinuities [19]. Quite surprisingly, we observe the subject’s daily rhythms to strongly influence the assignment of epileptic brain dynamics to either of these two classes which may account for the reported difficulties.

## 2. Materials and Methods

We begin with recalling the definition of a jump-diffusion process together with the relationships of its functions and parameters to KM coefficients. We then recall a criterion to distinguish diffusive and jumpy behavior in time series and illustrate its suitability using time series of diffusion and jump-diffusion processes.

### 2.1. Jump-Diffusion Modeling

With the Itô interpretation of a stochastic process [20], a typical jump-diffusion dynamics can be defined as [16,18,19]

(1) dx(t)=a(x,t) dt+b(x,t) dW(t)+ξdJ(t),

where {W(t),t≥0} is a scalar Wiener process, a(x,t) and b(x,t) are the state-dependent deterministic drift and the multiplicative diffusion functions, and J(t) is a time-homogeneous Poisson jump process (we assume that jump events are rare and can be modeled via a Poisson process). Jumps have state-dependent rate λ(x) (which defines the mean waiting time $\tau_{p}=1/\lambda$ between successive jumps) and size ξ, which we assume to be Gaussian distributed with zero mean and variance $\sigma_{\xi }^{2}$ (or to follow any symmetric distribution with finite moments).

For infinitesimal dt, it was shown in Ref. [16] that the functions and parameters of process (1) are related to the *m*-th order KM coefficients $D^{\left[m\right]}\left(x,t\right)=M^{\left[m\right]}\left(x,t\right)/m!=\lim_{dt\to 0}\;\frac{1}{m!dt}K^{\left[m\right]}\left(x,t,dt\right)$ as

(2) $$\begin{array}{ccc} K^{\left[1\right]}\left(x,t,dt\right) & = & a(x,t)\;dt,\\ K^{\left[2\right]}\left(x,t,dt\right) & = & \left[b^{2}\left(x,t\right)+\langle \xi^{2}\rangle \lambda \left(x\right)\right]\;dt,\\ K^{\left[2m\right]}\left(x,t,dt\right) & = & \langle \xi^{2m}\rangle \lambda \left(x\right)\;dt,\;\mathrm{for}2m>2. \end{array}$$

We note that $\langle \xi^{2j}\rangle =\frac{(2j)!}{2^{j}j!}{\langle \xi^{2}\rangle }^{j}=\frac{(2j)!}{2^{j}j!}$. From this combinatorial expression and with $\sigma_{\xi }^{2}=\langle {\left(\xi -\langle \xi \rangle \right)}^{2}\rangle =\langle {\left(\xi -0\right)}^{2}\rangle =\langle \xi^{2}\rangle$ two important model parameters can be deduced for a Gaussian-distributed random variable with zero-mean, namely the jump amplitude $\sigma_{\xi }^{2}$ and the jump rate λ:

(3) $$\sigma_{\xi }^{2}=\frac{M^{\left[6\right]}}{5M^{\left[4\right]}}\;\mathrm{and}\;\lambda =\frac{M^{\left[4\right]}}{3\sigma_{\xi }^{4}}.$$

Hence, it is possible to compute all jump-diffusion model parameters from the first six KM coefficients.

### 2.2. Distinguishing Diffusion from Jump-Diffusion Processes

In Ref. [19], a criterion was introduced that allows one to distinguish between diffusive and jump-diffusive behavior. If the investigated dynamics is purely diffusive, one finds

(4) $$K^{\left[4\right]}\left(x,t,dt\right)\approx 3{\left(K^{\left[2\right]}\left(x,t,dt\right)\right)}^{2}.$$

**Figure 1:**
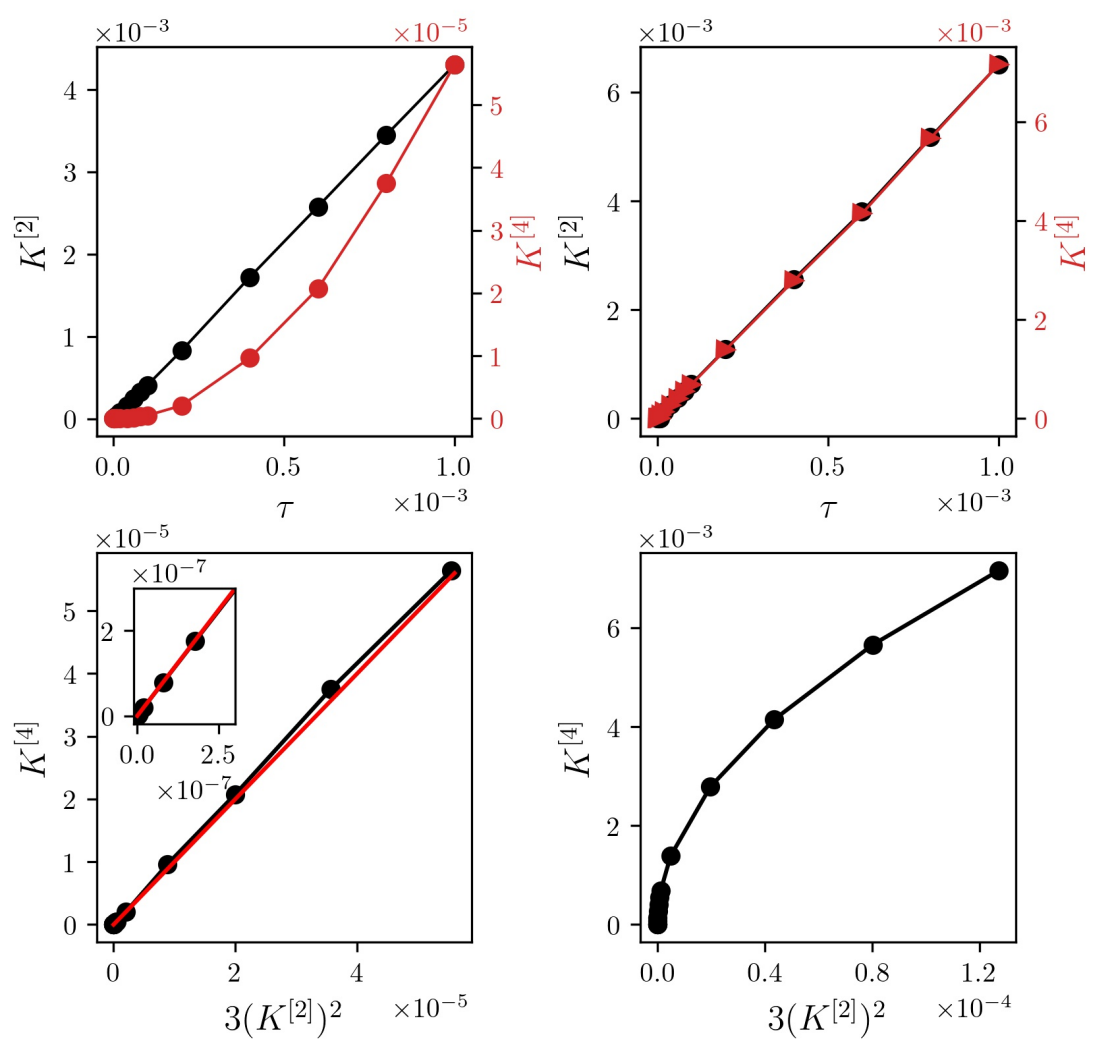
(**Top**) Dependencies of conditional moments $K^{\left[4\right]}$ (red) and $K^{\left[2\right]}$ (black) on time interval dt for exemplary time series of a continuous diffusion process (a(x)=−4x and b(x)=2; (**left**) and a jump-diffusion process (a(x)=−4x, b(x)=2, λ=3, $\sigma_{\xi }^{2}=1$; (**right**). Time series consisted of $N=10^{6}$ data points each (Euler-Maruyama integration scheme). (**Bottom**) $K^{\left[4\right]}$ versus $3{\left(K^{\left[2\right]}\right)}^{2}$ for the respective processes. Black lines are for eye-guidance only. The red line is the theoretical prediction.

This relationship cannot only separate jump-diffusion from diffusion processes, it can more importantly also determine whether a process is diffusive, even though numerically the fourth-order moment does not vanish (cf. Pawula theorem [21]). From Equation (4), one finds $K^{\left[2\right]}$ to increase linearly with dt, while $K^{\left[4\right]}$ scales with $dt^{2}$ for diffusive processes (cf. Figure 1).

While the linear relationship between second- and fourth-order conditional moment holds for pure diffusion processes [19], we observe a radicular relationship for the considered jump-diffusion process. Whether this holds for jump-diffusion processes in general would require further investigations.

### 2.3. Analysis of EEG Time Series Data

We analyzed EEG time series data that was recorded in a subject suffering from a drug-resistant focal epilepsy. In such cases, freedom of seizures can be obtained by resecting the part of the brain responsible for seizure generation (epileptic focus). Taking such sort of data is mandatory as part of the presurgical analysis. The sensoring electrodes were left in the brain for two weeks. During this time, the subject was also watched by video, so that EEG activity could be matched with behavior. The analyses reported here were made after surgery had taken place, and after it had become clear from its success whether the location of the epileptic focus had been correctly predicted. EEG was recorded from electrodes implanted under the skull, hence close to the epileptic focus and to other suspected brain regions and with high signal-to-noise ratio. Using a 16 bit analog-to-digital converter, the EEG signals were sampled at 200 Hz (sampling interval dt=5ms) and filtered with the frequency band 0.1–70 Hz.

For our investigations, we consider EEG time series data from two brain regions, namely from within the epileptic focus and from a distant, non-affected region of the opposite brain hemisphere. We split the EEG time series into consecutive non-overlapping windows of 50 s duration each (corresponding to $10^{4}$ data points). This window length can be regarded as a compromise between the required statistical accuracy for calculating the conditional moments and approximate stationarity of the data within a window [15]. For each window, we normalized the data to zero mean and unit variance and used a histogram-based approach [18] to calculate the coefficients $K^{\left[m\right]}\left(x,t,dt\right)=\langle {\left[x\left(t+dt\right)-x\left(t\right)\right]}^{m}\rangle {|}_{x(t)=s}$ within the range s∈[−2σ,2σ].

## 3. Results

**Figure 2:**
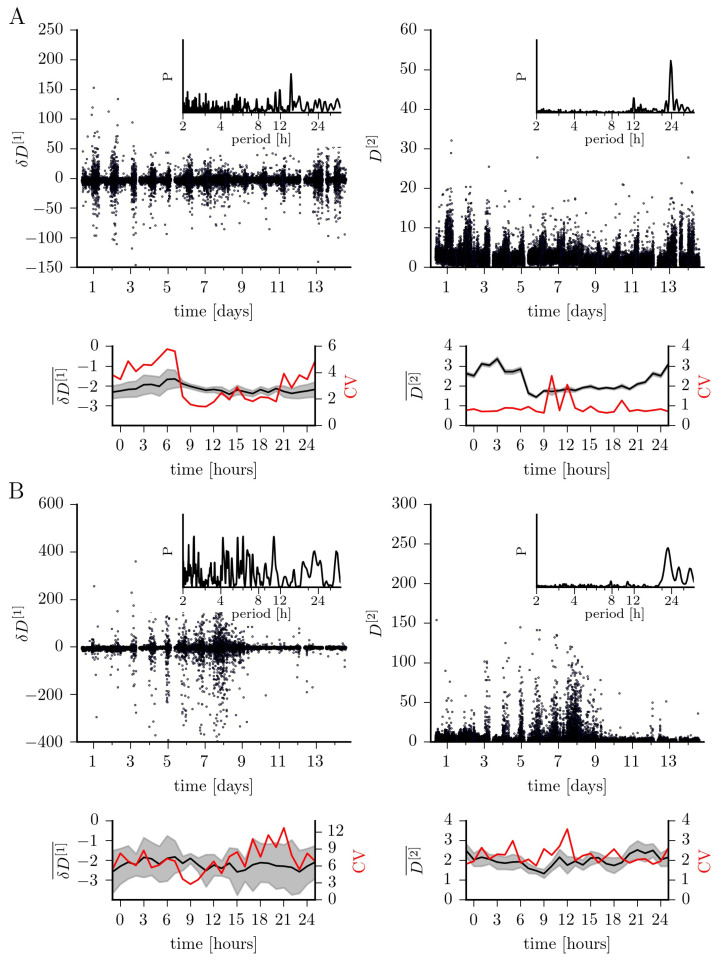
Fluctuations of the slope of the first-order Kramers–Moyal coefficient ($\delta D^{\left[1\right]}$; drift) and of the second-order coefficient ($D^{\left[2\right]}$; diffusion) over 14 days for exemplary EEG data from within the epileptic focus (**A**) and from a non-affected region (**B**). Discontinuities in the temporal evolutions are due to recording gaps, and tics on x-axes denote midnight. The coefficients’ medians ($\bar{\delta D^{\left[1\right]}}$, $\bar{D^{\left[2\right]}}$) and their standard error (grey-shaded areas) within a 24 h time period (estimated from non-overlapping windows of 1 h duration) as well as the medians’ coefficient of variation (CV) estimated from the 14 days. Lines are for eye-guidance only. Insets show normalized power spectral density estimates *P* (area under the curve equals 1) [26] of the respective temporal evolutions demonstrating ultradian (less than 24 h) and circadian (around 24 h) peaks in periodicity as well as infradian contributions (larger than 24 h).

In Figure 2, we show how the first two conditional moments fluctuate over a period of 14 days and within a time period of 24 h. Confirming earlier studies [13,16], we observe the slope of the drift coefficient ($\delta D^{\left[1\right]}$) to indicate an overall linear damping behavior (epileptic focus (median ± std. dev.): −2.2±8.8; non-affected brain region: −2.2±28.1). In order to gain further insights, we group the recording into those taken during daytime (6 a.m. to 10 p.m.) and those taken at nighttime (10 p.m. to 6 a.m.; since no sleep-scoring was available for this subject, we cannot evaluate the influence of different sleep stages). Median values of $\delta D^{\left[1\right]}$ for the epileptic focus compare to the ones obtained for the non-affected brain region both for recordings taken at daytime (epileptic focus: −2.2±6.8; non-affected brain region: −2.2±28.4) and at nighttime (epileptic focus: −2.1±12.5; non-affected brain region: −2.2±27.3). The four-fold lower variability of $\delta D^{\left[1\right]}$ for recordings taken at daytime from the epileptic focus is enhanced by a factor of two for recordings taken at nighttime, which can probably be attributed to sleep-induced nonlinearities [22,23,24]. There are additional strong fluctuations around day 7; these might be due to a modification of the dose of the antiseizure medication, which is sometimes done in order to increase the probability for seizures, and is known to alter nonlinearities in the human epileptic brain dynamics [25].

We observe similar time-dependent fluctuations for the diffusion coefficient. Overall, it takes on similar median values for the dynamics from both brain regions, however, with strongly enhanced variability for the non-affected brain region (epileptic focus: 2.1±2.7; non-affected brain region: 2.0±10.5). While we obtain comparable data for recordings taken at daytime (epileptic focus: 1.9±2.5; non-affected brain region: 1.9±10.3), the median diffusion coefficient for the dynamics from within the epileptic focus is clearly enhanced for recordings taken at nighttime (epileptic focus: 2.8±2.7; non-affected brain region: 2.1±10.8). More importantly, in addition to the aforementioned influencing factors, circadian and probably even infradian rhythms strongly impact on the fluctuations seen for the diffusion coefficient.

**Figure 3:**
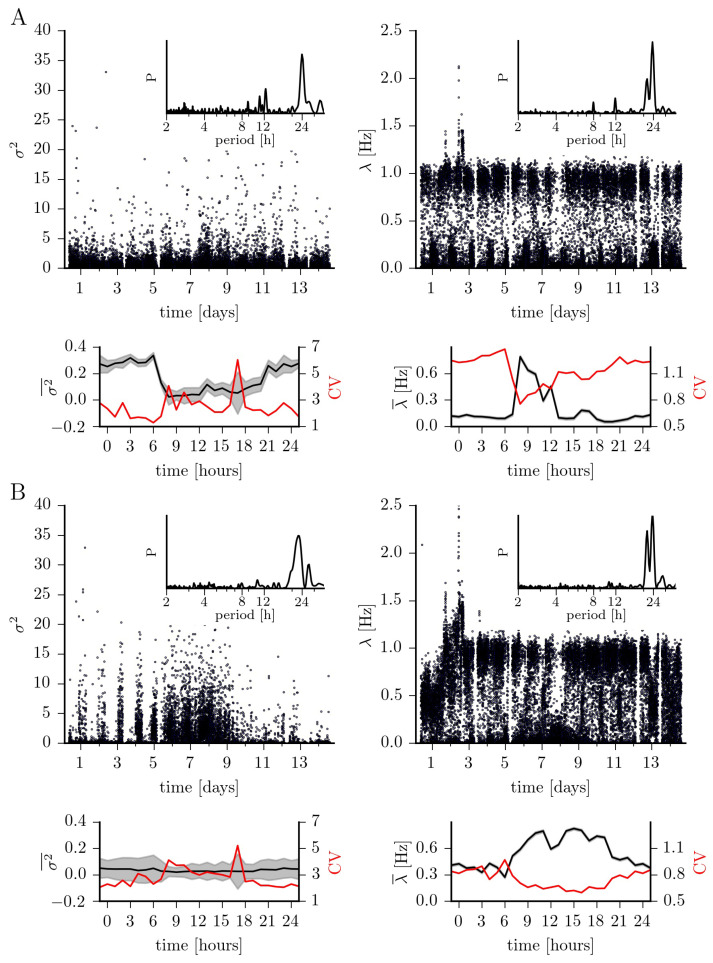
Same as Figure 2 but for jump amplitude $\sigma_{\xi }^{2}$ and jump rate λ.

In Figure 3, we present our findings for the jump characteristics (amplitude and rate). Jump amplitudes differ, in general, by a factor of six between brain regions (epileptic focus: 0.2±1.8; non-affected brain region: 0.03±2.3). For the epileptic focus, the temporal evolution is, however, strongly affected by the circadian rhythm with median jump amplitudes for recordings taken at daytime of 0.1±1.9 increasing to 0.3±1.2 for recordings taken at nighttime. For the non-affected brain region, median jump amplitudes for daytime-data (0.03±2.2) compare to those seen for nighttime-data (0.04±2.6).

The jump rates for the dynamics within the epileptic focus and from the non-affected brain region appear to be bimodally distributed. At closer inspection one can however notice that for the epileptic focus this seeming bimodality is dominated by circadian and ultradian rhythms: for recordings taken at nighttime, we observe the jump rate to be rather low (0.1±0.3 Hz), in general; for recordings taken at daytime in the mornings the jump rate is about fivefold higher (0.5±0.4 Hz) but in the afternoons it compares to the one seen for nighttime-data (0.1±0.4 Hz). For the dynamics from the non-affected brain region, the seeming bimodality is dominated by the circadian rhythm only, with median jump rates for nighttime-data being about 75% higher than those for daytime-data (0.7±0.4 Hz vs. 0.4±0.4 Hz).

**Figure 4:**
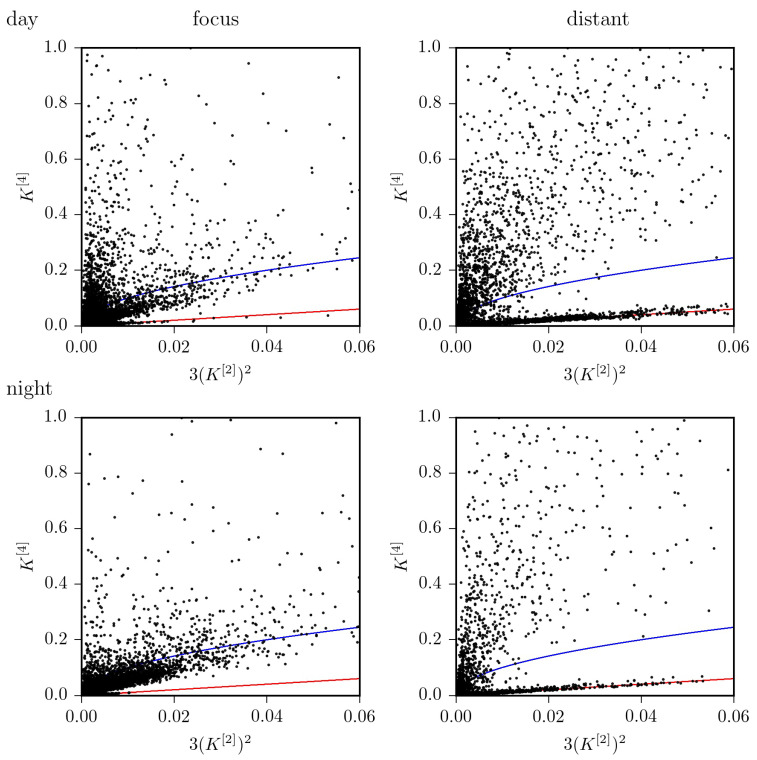
$K^{\left[4\right]}$ versus $3{\left(K^{\left[2\right]}\right)}^{2}$ for all EEG data segments from recordings taken at daytime (**top**) and at nighttime (**bottom**) from within the epileptic focus (**left**) and from a distant, non-affected brain region (**right**). The identity line is shown in red; the radicular relationship (see Figure 1) is shown in blue.

**Figure 5:**
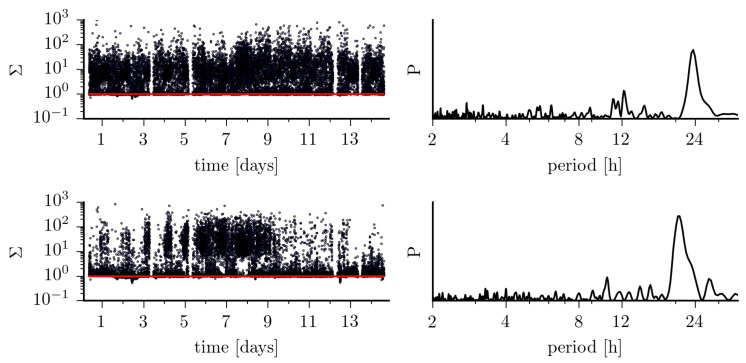
(**Left**) Fluctuations of the ratio $\Sigma =K^{\left[4\right]}/3{\left(K^{\left[2\right]}\right)}^{2}$ over 14 days for EEG data from within the epileptic focus (**top**) and from a non-affected region (**bottom**). The red lines indicate a purely diffusive dynamics (Σ=1). (**Right**) normalized power spectral density estimates *P* of the respective temporal evolutions.

Findings achieved so far, indicate that—on average—the jump-diffusion modeling appears better suited to improve characterization of pathological brain dynamics [16], while the Langevin-type modeling may be sufficient for a characterization of physiological activities [13]. However, the strong impact of ultradian, circadian as well as of infradian rhythms on higher-order conditional moments of epileptic brain dynamics impinges crucially on the choice of the model. Using Equation (4), we find that the dynamics of the distant, non-affected brain region can be considered as purely diffusive in only about 42% of the total observation time. For the dynamics of the epileptic focus this holds for 33% of the total observation time. If we consider recordings taken at daytime, these figures increase to 47%, resp. 38%, but drop to 29%, resp. 19% for recordings taken at nighttime (cf. Figure 4 and Figure 5). Of the remaining percentages, only a small fraction of data (epileptic focus: 2.9%; non-affected brain region: 0.6% of total observation time) is in line with the radicular relationship between $K^{\left[4\right]}$ and $3{\left(K^{\left[2\right]}\right)}^{2}$ mentioned above for the jump-diffusion process considered here, along with the choice of functions and parameters. This may indicate the need to consider other forms of jump-diffusion processes.

## 4. Conclusions

Time series of observables from complex dynamical systems often exhibit fluctuations together with jump discontinuities between different system states. Among others [18], such a jump-diffusion behavior was reported recently for pathological dynamics of the human brain (epilepsy [16]) and heart (congestive heart failure [27]). Since the normal, physiologic dynamics of these organ systems often appear as continuous stochastic processes, a time-series-analysis-based distinction between jumpy and diffusive dynamics using higher-order Kramers-Moyal coefficients [19] would, in principle, allow one to distinguish healthy and diseased states. However, given that such open, dissipative, and adaptive dynamical systems are inherently nonstationary [8,28,29,30], a clear-cut distinction cannot be expected. Rather, stochastic properties of their dynamics will depend on time, which may lead to a switching between diffusive and jumpy behavior [27]. Our findings indicate that such a switching may be due to an exogenous and/or endogenous forcing, expressing itself as ultradian, circadian, and even infradian rhythms [31]. If similar observations can be made in a larger group of subjects, future studies would need to take the influence of such forcings into account when constructing evolution equations for such complex dynamical systems. 

## Data Availability

The data that support the findings of this study are available from the corresponding author upon reasonable request. The data are not publicly available as they contain information that could compromise the privacy of research participants.
